# Effect of Citric Acid and Ethylenediaminetetraacetic Acid on the Surface Morphology of Young and Old Root Dentin

**DOI:** 10.7508/iej.2016.03.008

**Published:** 2016-05-01

**Authors:** Miriam Zaccaro Scelza, Fernando de Noronha, Licinio Esmeraldo da Silva, Marcos Maurício, Marco Antonio Gallito, Pantaleo Scelza

**Affiliations:** a *Department of Endodontics, Fluminense Federal University (UFF), Niteroi, Rio de Janeiro, Brazil; *; b * Department of Materials Engineering, Pontifical Catholic University of Rio de Janeiro (PUC), Rio de Janeiro, Brazil *

**Keywords:** Citric Acid, Dentinal Tubule, Ethylenediaminetetraacetic Acid, Scanning Electron Microscopy, Surface Morphology

## Abstract

**Introduction:**

The aim of this *in vitro* study was to evaluate the effect of 10% citric acid and 17% ethylenediaminetetraacetic acid (EDTA) irrigating solutions on the surface morphology of young and old root dentin by determining the number and diameter of dentinal tubules using scanning electron microscopy (SEM).

**Methods and Materials:**

Fifty healthy human teeth collected from young (≤30 years) and old (≥60 years) individuals (*n*=25) were first prepared with a Largo bur #2 to produce smear layer on the root canal surface. Subsequently, the crowns and the root middle and apical thirds were sectioned and removed, and the cervical thirds were sectioned vertically in the buccal-lingual direction into two equal halves. The obtained samples were then immersed in 2.5% sodium hypochlorite for 30 min and randomly separated into two treatment groups for each age group. In each age group, ten samples were selected as controls and did not receive any type of treatment. The rest of the specimens were then rinsed, dried and treated for 4 min with 10% citric acid or 17% EDTA. The samples were then assessed with SEM regarding the number and diameter of dentinal tubules. All data were assessed using Student’s t-test. The level of significance was set at 0.05.

**Results:**

Regardless of the type of treatment, no significant differences were observed in the number of open tubules between the young and old root dentin (*P*>0.05). Nonetheless, the diameter of the tubules in the old root dentin was larger when 17% EDTA was used (*P*<0.05). Both, young and old root dentin did not differ with the 10% citric acid treatment (*P*>0.05).

**Conclusion:**

The results showed that 17% EDTA treatment induced a significant demineralization in old root dentin.

## Introduction

Due to the improvements in the quality of oral health a large number of fully dentate adults are present that need routine dental treatments [1]. An outcome attributed to the increase of the number of dentate adults is the occurrence of crown and root caries as high as 65% in patients exceeding 65 years of age [2]. In these patients, endodontic treatment is sometimes necessary.

Dentin is a hydrated tissue that comprises of an extensive collection of tubules with odontoblast extensions that spread to the pulp tissue. Dentinal tubules are delimited by a hypermineralized peritubular dentin and a less mineralized intertubular dentin [3]. Chemical and physical modifications of dentin related to age are characterized by the continuous deposition of peritubular dentin, resulting in decreased number of dentin tubules [4]. 

During root canal instrumentation, smear layer is formed which is an amorphous structure composed of inorganic and organic materials and is 1-2 µm thick on average, although it might fill dentinal tubules in thicknesses of up to 40 µm. It has been reported that smear layer holds bacteria or bacterial products and may act as a reservoir for irritants, which would indicate its removal [5-7]. 

Various chemical substances are employed in endodontic treatment which are capable of removing the smear layer and modifying the structural and chemical composition of the root dentin, resulting in changes in its permeability and solubility.

And amongst these irrigants are citric acid and ethylenediaminetetraacetic acid (EDTA) [8, 9]. Citric acid is a decalcifying and cleansing solution [10]. EDTA, in combination with sodium hypochlorite, can also enhance the removal of smear layer [10], promoting a progressive dissolution at the cost of erosion in peritubular and intertubular dentin [11].

The purpose of this *in vitro* study was to evaluate the effects of 10% citric acid and 17% EDTA solution on young and old root dentin by determining the number and diameter of open dentinal tubules in the cervical third fragments of root, using scanning electron microscopy (SEM).

## Materials and Methods

The study protocol was registered under protocol number of 01790312.5.0000.5243 CAAE. Fifty human mandibular first premolars that were extracted for therapeutic reasons were collected from the Human Tooth Bank at the Faculty of Dentistry of the Fluminense Federal University, Rio de Janeiro, Brazil. 

Immediately after extraction, the teeth were stored in distilled water. They were collected from young (≤30 years) and old (≥60 years) patients (*n*=25). The criteria for tooth selection included the absence of caries, restorations, cracks or fractures in either the root or crown. 

**Figure 1:**
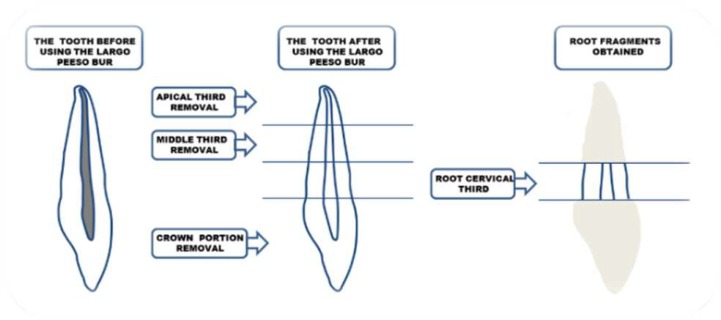
Specimen preparation process

The crown and the middle and apical thirds of the roots were transversely sectioned and removed using double-sided diamond discs (KG Sorensen, Cotia, SP, Brazil) so that the root cervical thirds were remained. For specimen preparation, a #2 Largo Peeso Reamer (Dentsply Maillefer, Ballaigues, Switzerland) was first used to prepare the lumen of the canals and produce a smear layer in the root canals of all teeth, except for the control group. Subsequently, the root cervical thirds were once again vertically sectioned in the bucco-lingual direction, dividing each sample into two equal halves (Figure 1). In addition, to reduce variables, preparation of all sample was performed by the same operator. The obtained samples were then immersed in 2.5% sodium hypochlorite for 30 min.

Then, the root fragments were rinsed, dried and treated with test solutions for 4 min as follows: *Group Y1*; twenty fragments of young root dentin were treated with 10% citric acid (Fórmula & Ação Farmácia, São Paulo, SP, Brazil); *Group Y2*; twenty fragments of young root dentin were treated with 17% EDTA (Fórmula & Ação Farmácia, São Paulo, SP, Brazil); *Group O1*; twenty fragments of old root dentin were treated with 10% citric acid; *Group O2*; twenty fragments of old root dentin were treated with 17% EDTA. Moreover, in each group, 10 fragments were selected as controls and did not receive any solution treatment. 

The samples were then dried, placed in a vacuum chamber and coated with a 20-nm-thick gold layer for SEM evaluation (JSM 6510 LV, Japan Electron Optics Laboratory Co., JEOL Ltd, Osaka, Japan). SEM images were obtained using an acceleration tension of 10 kVp in the secondary electron (SE) and back-scattered (BSE) electron modes, at ×1500 magnifications. The measurements of dentinal tubules were performed using AxioVision 4.8 software (Carl Zeiss MicroImaging GmbH, Jena, Germany). A typical image processing and analysis sequence was applied to each SE-BSE image, which involved the main steps of pre-processing, segmentation, post-processing, measurement and data analysis [12, 13]. In the pre-processing step, a sigma filter was applied to reduce the noise that is typically present in SE-BSE images and to preserve the dentinal tubule edges. In the segmentation step, the dentinal tubules were discriminated by applying a threshold to the gray level intensity. The segmentation result was a binary image containing the objects of interest, white regions and a black background. Post-processing was used to avoid segmentation problems, including residual particles dispersed in the dentine and fragmented objects that could be mistakenly measured as small tubules. Very small objects were removed using an area criterion (scrap function), and fragmented tubules were put together using a close function. All images were analyzed, and field parameters related to human dentin characterization were obtained in significant statistical quantities. In each image, the means of the field measurements were analyzed for the following parameters: number of tubules per unit area (density) and diameter (circle with equivalent area) of dentinal tubules.

The number of taken images was different for each sample. Student’s t-test was used for the statistical analysis. The analyses was done using the SPSS software (SPSS version 18, IBM, Chicago, IL, USA) and the level of significance was set at 0.05.

## Results

According to the age groups, the mean±SD of the diameter and number of open dentinal tubules per square mm are presented in Table 1. When comparing the number of dentinal tubules independent of age, no significant differences were observed between the 10% citric acid and 17% EDTA treatments (*P*>0.05). However, statistically significant differences were observed when using 17% EDTA solution in the old dentin group, indicating more enlargement of the dentinal tubule diameter compared to the young dentin group (*P*<0.05).

**Figure 2:**
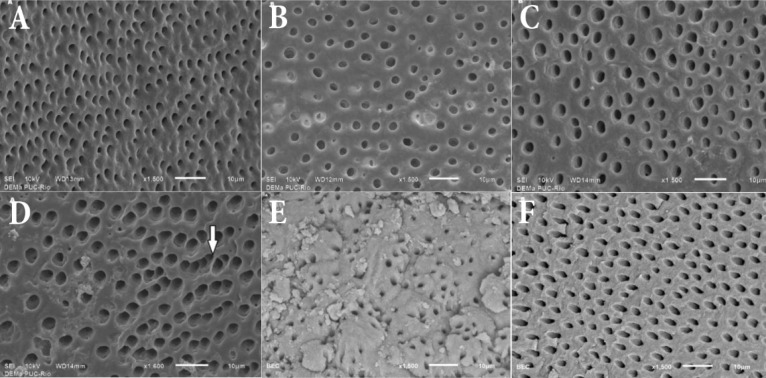
Representative photomicrographs of the surface of: *A)* Young root dentin treated with 10% citric acid (Group Y1) presents increased dentinal tubule numbers compared to the old dentin; *B)* Old root dentin treated with 10% citric acid (Group O1); *C)* Young root dentin after the use of EDTA (Group Y1); *D)* Old root dentin after the use of EDTA with an irregular outline of dentinal tubules (arrow) (Group Y2). Root dentin surface of the control samples; *E)* Shows the old dentin tubules with a reduced diameter compared with the young dentin; *F)* ×1500 magnification

## Discussion

Dentin makes most of the structure of human teeth and is composed of peritubular and intertubular dentin. Dentinal tubules extend through the entire dentin thickness but vary both in number and diameter [14]. It has been reported that the diameter and number of dentinal tubules influence the adhesion of dental material, as they are related to dentinal permeability [15]. Natural changes that occur during aging, affect the ultra-morphological characteristics of dentin, including the number and conformation of dentinal tubules [16]. The present study aimed to investigate the effects of 10% citric acid and 17% EDTA solutions on young and old root dentin by determining the number and diameter of dentinal tubules in root fragments after being in contact with either of these two solutions.

Considering the treatment time of dentinal surface, with the test substances, the literature has reported a 4-min contact time as the most efficient to remove the smear layer [17].

In this work, although there was no statistically significant difference when comparing the number of the tubules between the age groups, the number of open dentinal tubules was lower in old root dentin compared to young root dentin when 10% citric acid was used. This finding could be explained by the continuous deposition of mineralized tissue in peritubular dentin [18]. The number of dentinal tubules per unit area (density) decreases with age [19]. The literature has previously reported a reduction in the diameter of tubule lumens in specimens obtained from elderly patients showing that the extent of occlusion increases with age [20], which was corroborated in the control group in the present investigation (Figure 2). 

When comparing the age groups after using the different test substances, statistically significant differences (*P*<0.05) were found following the use of 17% EDTA in the old dentin group indicating an increase in the diameter of dentinal tubules. This result has been explained by an increase in the mineralized tissue on old root dentin that is caused by a stronger chelating activity of 17% EDTA [21].

By analyzing the number of dentinal tubules independent of age, the present research showed no difference between the 10% citric acid and 17% EDTA treatments used for 4 min. These results corroborate the findings of the study by Scelza *et al.* [21], which reported no significant difference between the tested substances (10% citric acid, 17% EDTA and EDTA-T), when teeth were treated for 3 min [22]. 

Another point of discussion regarding the changes in the number of tubules is related to the root region analyzed. This research evaluated only dentinal tubules in the cervical third area of the root because they have been reported to have a significantly greater numerical density of tubules than the other root thirds [23, 24]. 

**Table 1.** Mean (SD) of the number and diameter of open dentinal tubules per mm^2^ according to the type of irrigation and group of age (^a^ *P*<0.05

**Treatment**	**Open dentinal tubules (N/mm** ^2^ **) (N=20)**		**Diameter of tubules (µm) (N=20)**	
	**Old root **	**Young root **	**Old root **	**Young root **
**Citric acid**	122 (14)	152 (19)	2.0 (0.1)	1.7 (0.2)
**EDTA**	161 (22)	140 (17)	2.3 (0.1)	1.7 (0.2) ^a^
**Control**	147 (10)	159 (24)	1.6 (0.2)	1.9 (0.2)

In the present investigation, SEM was used to quantify the number and diameter of dentinal tubules after treatment. SEM image and parametric estimation are the most commonly used technique to evaluate the number of tubules (density) in sections of demineralized or under-mineralized dentin [24-26].

Image investigations of dentinal tubules indicated that old dentin has more shape irregularities than young dentin, regardless of the solution used. This evidence has supported the argument that higher mineralization results in greater changes to the outline of tubules after treatment; thus, filling material adhesion to peritubular dentin could be hindered by the difficulty involved in demineralizing the surface, leading to less formation of "tags" [27].

## Conclusion

No significant differences were observed in the number of open tubules between the young and old root dentin after using the irrigating solutions. On the other hand, the diameter of the tubules in the old root dentin was larger when 17% EDTA was used, suggesting a significant demineralization in old root dentin. Young and old root dentin did not differ with the 10% citric acid treatment.
